# Prescribing patterns and medication costs in patients on maintenance haemodialysis and peritoneal dialysis

**DOI:** 10.1093/ndt/gfae154

**Published:** 2024-07-04

**Authors:** Anukul Ghimire, Anita M Lloyd, Aminu K Bello, Marisa Battistella, Paul Ronksley, Marcello Tonelli

**Affiliations:** Department of Medicine, University of Calgary, Calgary, AB, Canada; Department of Medicine, University of Alberta, Edmonton, AB, Canada; Department of Medicine, University of Alberta, Edmonton, AB, Canada; University Health Network, University of Toronto, Toronto, ON, Canada; Department of Medicine, University of Calgary, Calgary, AB, Canada; Department of Medicine, University of Calgary, Calgary, AB, Canada

**Keywords:** drug interactions, haemodialysis, kidney failure, medications, peritoneal dialysis

## Abstract

**Background:**

Polypharmacy is a significant clinical issue for patients on dialysis but has been incompletely studied. We investigated the prevalence and costs of polypharmacy in a population-based cohort of participants treated with haemodialysis (HD) or peritoneal dialysis (PD).

**Methods:**

We studied adults ≥20 years of age in Alberta, Canada receiving maintenance HD or PD as of 31 March 2019. We characterized participants as users of 0–29 drug categories of interest and those ≥65 years of age as users/non-users of potentially inappropriate medications (PIMs). We calculated the number of drug categories, daily pill burden, total annual cost and annual cost per participant and compared this to an age- and sex-matched cohort from the general Alberta population.

**Results:**

Among 2248 participants (mean age 63 years; 39% female) on HD (*n* = 1781) or PD (*n* = 467), the median number of prescribed drug categories was 6 [interquartile range (IQR) 4–8] and the median daily pill burden was 8.0 (IQR 4.6–12.6), with 5% prescribed ≥21.7 pills/day and 16.5% prescribed ≥15 pills/day. Twelve percent were prescribed at least one drug that is contraindicated in kidney failure. The median annual per-participant cost was ${\$}$3831, totalling ≈${\$}$11.6 million annually for all participants. When restricting to the 1063 participants ≥65 years of age, the median number of PIM categories was 2 (IQR 1–2), with a median PIM pill burden of 1.2 pills/day (IQR 0.5–2.4). Compared with PD participants, HD participants had a similar daily pill burden, higher use of PIMs and higher annual per-participant cost. Pill burden and associated costs for participants on dialysis were >3-fold and 10-fold higher, respectively, compared with the matched participants from the general population.

**Conclusion:**

Participants on dialysis have markedly higher use of prescription medications and associated costs than the general population. Effective methods to de-prescribe in the dialysis population are needed.

## Graphical Abstract

**Graphical Abstract:**
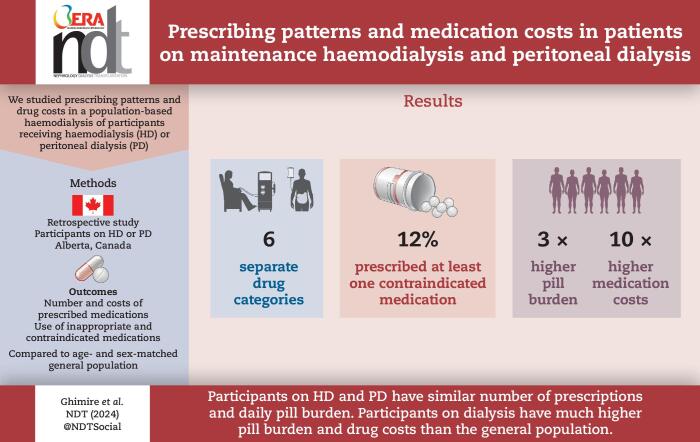

KEY LEARNING POINTS **What was known:**

- Polypharmacy and the use of potentially inappropriate medications (PIMs) are linked to patient harm and increased healthcare expenditures.
- Most data about polypharmacy in patients on dialysis is derived from in-centre haemodialysis (HD) cohorts and does not contain information on the use of contraindicated medications.
- Limited data describe the prevalence and costs of polypharmacy in patients on HD or peritoneal dialysis (PD) and those without kidney failure.

**This study adds:**

- In a population-based cohort of 2248 participants on maintenance HD or PD, participants were prescribed a median of six separate drug categories and two PIM categories and 12% were prescribed a contraindicated medication. The median daily pill burden was 8.
- Despite similar rates of prescribed medications and daily pill burden, participants on HD had much higher medication costs than those on PD, largely related to the use of erythropoietin stimulating agents.
- Participants on dialysis had a >3-fold higher rate of pill burden and 10-fold higher medication costs compared with age- and sex-matched controls from the general population.

**Potential impact:**

- The median number of prescribed medications is similar for participants on maintenance HD and PD but pill burden and medication costs are markedly higher for dialysis participants than the general population.
- The use of PIMs and contraindicated medications was relatively common in participants on HD or PD.
- Effective strategies for de-prescription in the dialysis population are needed.

## INTRODUCTION

Polypharmacy is defined as the simultaneous use of five or more medications [1, 2] and has been identified by the World Health Organization as a notable cause of patient harm [3]. Patients with kidney failure treated with haemodialysis (HD) are typically prescribed 8–12 separate medications [4–7], with a daily burden of 19 pills [8]. Higher pill burden is an independent risk factor for mortality and all-cause hospitalization in patients on maintenance dialysis [9, 10] and polypharmacy is a major cost driver in this population [4]. Further, an increasing number of medications may be needed as patients age, develop additional comorbidities and accumulate time on dialysis. Although patients on HD often require treatment for diabetes, hypertension and cardiovascular disease, a significant proportion of their prescriptions are considered potentially inappropriate medications (PIMs) [11–16]. These medications increase the risk of adverse events, decrease quality of life and increase healthcare costs [1, 2, 6, 8, 17–20].

Current knowledge about polypharmacy and PIM use in dialysis populations is based on studies of in-centre HD units. Fewer data describe polypharmacy in patients treated with maintenance peritoneal dialysis (PD), who tend to have different baseline characteristics [21], better clinical outcomes [22] and perhaps fewer comorbidities than patients treated with maintenance HD [7, 11, 23], due in part to selection bias favouring younger patients with less comorbidity [24]. Very few studies have compared prescribing patterns between patients on PD and HD or compared medication use among patients with kidney failure with an otherwise comparable population who is free of kidney failure [7]. Finally, few studies have systematically evaluated the frequency with which medications that are contraindicated in kidney failure are prescribed in this population.

We designed this study to analyse the burden of polypharmacy in an unselected population-based sample of participants treated with maintenance HD or PD in Alberta, Canada. We aimed to describe the prevalence and costs of polypharmacy, prescription of PIMs and contraindicated medications, differences in prescribing patterns for participants treated with HD compared with PD and differences in prescribing patterns and medication costs between participants on dialysis and an otherwise comparable population without kidney failure.

## MATERIALS AND METHODS

### Population and data sources

We used the Alberta Kidney Disease Network (AKDN) database, a previously described population-based database [25] that incorporates data from Alberta Health (AH; the provincial health ministry), including demographics, provider claims, hospitalizations and ambulatory care utilization; the Northern and Southern Alberta Kidney Care programs (AKC-N and AKC-S) and the clinical laboratories in Alberta. In addition to these data sources, we also used the Alberta Pharmaceutical Information Network (PIN), which captures prescription drug information on all medications dispensed in Alberta. More than 99% of Alberta residents (population 4.3 million people) are registered with Alberta Health and have universal access to hospital care and physician services. The institutional research ethics boards at the Universities of Calgary and Alberta approved the study (REB16-1575, psite00000147) with a waiver of individual signed patient consent (since data were de-identified).

We used these linked data sources to assemble a cohort of participants who were receiving chronic HD or PD as of 31 March 2019 (index date) and had been receiving dialysis since at least 1 December 2018 (i.e. ≥120 days). We excluded individuals who switched dialysis modalities within 120 days on or prior to the index date, did not have at least one medication dispensed within 120 days of the index date or were <20 years of age as of the index date. The latter exclusion allowed for a look-back of at least 2 years for comorbidity assessment, since the AKDN database includes laboratory, hospitalization and claims data for participants ≥18 years of age. Dialysis vintage was calculated as the time spent on the most recent modality of dialysis (as of index date) and did not include time on any previous dialysis modalities. We also assembled a cohort of participants without kidney failure (i.e. controls; those who were not currently or previously receiving dialysis nor had a kidney transplant before 31 March 2019) who had at least one prescription (of any kind) in the PIN within 120 days of the index date. These participants were matched 10:1 on age (±2.5 years) and sex with those receiving HD or PD but were not matched on comorbidities. There were no restrictions placed on estimated glomerular filtration rate or proteinuria for inclusion in the control cohort.

### Ascertainment of medications use

We adapted a previously used approach [4] to classify medications as belonging or not to one of 29 drug categories as follows: allopurinol, alpha-1 blockers, angiotensin-converting enzyme (ACE) inhibitors, angiotensin receptor blockers, anticonvulsants, antidepressants, typical antipsychotics, prescription aspirin, benzodiazepines/hypnotics, beta blockers, bone health medications, bowel prokinetics, calcium channel blockers, digoxin, diuretics, dopamine agonists, fibrates, H2 receptor antagonists, oral hypoglycaemics, insulin, levodopa, prescription non-steroidal anti-inflammatory drugs (NSAIDs) other than aspirin, opioids, proton pump inhibitors (PPIs), statins, tamsulosin, vitamin D or vitamin D analogues and warfarin [4]. We also considered a miscellaneous category that was comprised of other drugs that were used by >10 participants within 120 days of the index date. Details of each study drug category are shown in Supplementary Table S1. We identified participants as users of a medication if there was evidence of at least one prescription dispensed within 120 days of the index date, based on the provincial PIN database.

### Ascertainment of contraindicated medications in kidney failure

We assessed the use of contraindicated medications using data from previous work [26–28]. We classified medications as being contraindicated in kidney failure if one or more of the cited studies suggested avoiding them in patients with an estimated glomerular filtration rate <15 ml/min/1.73 m^2^, creatine clearance <15 ml/min or ‘severe renal impairment’. We categorized medications into subcategories of anticoagulants, anticonvulsants, antihyperlipidaemics, anti-infectives, antineoplastics, anti-Parkinson’s, bisphosphonates, dementia medications, hypoglycaemics, NSAIDs other than aspirin, psychotherapeutic agents and ‘other’ (Supplementary Table S2).

### Ascertainment of potentially inappropriate medications

We assessed participants ≥65 years of age on the index date as users or non-users of a PIM using the Beers criteria [1] (Supplementary Table S3), also based on one or more prescriptions dispensed within 120 days of the index date.

### Ascertainment of baseline characteristics

We assessed baseline characteristics on the index date of 31 March 2019. We used postal codes to determine the Pampalon index of material deprivation [29, 30] (which categorizes participants into five categories of socio-economic inequalities in healthcare services and population health, with 5 representing the most deprived neighbourhoods), as well as rural versus urban location of residence. We used validated algorithms based on claims and hospitalization data to determine the baseline presence of 29 comorbidities [31]. We classified each participant with respect to the presence or absence of these 29 chronic conditions at the index date (look-back extended as far as April 1994 where records were available). Detailed methods for classifying comorbidity status and the specific algorithms used are found elsewhere [31].

### Statistical analyses

We reported baseline descriptive and medication use characteristics as counts and percentages or medians and interquartile ranges (IQRs), as appropriate. To assess differences between HD and PD groups, we used the Mann–Whitney U test for continuous variables and the χ^2^ or Fisher’s exact test for categorical variables. We calculated annual per-participant costs for each drug category in the year prior to 31 March 2019 by multiplying the quantity dispensed by the unit price for each dispensed drug. We obtained unit prices from archived versions of the Alberta Drug Benefit List from Alberta Health. We reported costs in Canadian dollars after inflating to 2021 costs using the consumer price index. All statistical analyses were done using Stata MP 18 (StataCorp, College Station, TX, USA).

## RESULTS

### Participants

Cohort creation is shown in Supplementary Fig. S1. There were 2248 participants receiving either HD or PD on 31 March 2019 and who had at least one study medication of interest dispensed during the 120 days on or prior to 31 March 2019. Table 1 shows the baseline characteristics of the cohort as of the index date (31 March 2019). Dialysis participants were on average 63 years old and were 39% female; 79% were receiving HD. Approximately 13% lived in a rural location and almost 50% were in the worst or second-worst material deprivation quintile. The most common comorbidities included hypertension (95%), diabetes (62%), chronic heart failure (45%) and peripheral vascular disease (31%). The median dialysis vintage for all participants was 2.4 years (IQR 1.1–4.7).

**Table 1:** Baseline characteristics of participants.

Characteristics	Dialysis participants (HD and PD) (*n* = 2248)	Dialysis participants ≥65 years of age (*n* = 1063)
Age (years), *n* (%)		
Mean (SD)	62.5 (15.0)	75.0 (7.0)
Median (IQR)	64.1 (53.4–72.9)	73.7 (69.1–80.0)
20–39.9	211 (9.4)	–
40–49.9	239 (10.6)	–
50–59.9	446 (19.8)	–
60–64.9	289 (12.9)	–
65–69.9	319 (14.2)	319 (30.0)
70–74.9	259 (11.5)	259 (24.4)
75–79.9	217 (9.7)	217 (20.4)
80–84.9	166 (7.4)	166 (15.6)
85–89.9	74 (3.3)	74 (7.0)
≥90	28 (1.3)	28 (2.6)
Female, *n* (%)	873 (38.8)	410 (38.6)
Rural residence location, *n* (%)	287 (12.8)	111 (10.4)
Residence in long-term care facility, *n* (%)	171 (7.6)	109 (10.3)
Material deprivation, *n* (%)		
1 (least deprived)	246 (10.9)	116 (10.9)
2	342 (15.2)	176 (16.6)
3	372 (16.6)	166 (15.6)
4	475 (21.1)	233 (21.9)
5 (most deprived)	616 (27.4)	269 (25.3)
Missing	197 (8.8)	103 (9.7)
HD, *n* (%)	1781 (79.2)	866 (81.5)
Comorbidities, *n* (%)		
Hypertension	2137 (95.1)	1030 (96.9)
Diabetes	1402 (62.4)	752 (70.7)
Chronic heart failure	1019 (45.3)	570 (53.6)
Peripheral vascular disease	692 (30.8)	359 (33.8)
Chronic pulmonary disease	666 (29.6)	390 (36.7)
Stroke and/or TIA	544 (24.2)	309 (29.1)
Chronic pain	430 (19.1)	204 (19.2)
Atrial fibrillation	411 (18.3)	270 (25.4)
Depression	371 (16.5)	147 (13.8)
Hypothyroidism	370 (16.5)	197 (18.5)
Myocardial infarction	262 (11.7)	167 (15.7)
Alcohol misuse	203 (9.0)	61 (5.7)
Epilepsy	163 (7.3)	68 (6.4)
Severe constipation	163 (7.3)	89 (8.4)
Cancer, non-metastatic	160 (7.1)	103 (9.7)
Asthma	155 (6.9)	63 (5.9)
Dementia	153 (6.8)	119 (11.2)
Rheumatic disease	144 (6.4)	66 (6.2)
Cancer, lymphoma	66 (2.9)	39 (3.7)
Cirrhosis	58 (2.6)	26 (2.5)
Cancer, metastatic	55 (2.5)	28 (2.6)
Inflammatory bowel disease	53 (2.4)	22 (2.1)
Psoriasis	52 (2.3)	29 (2.7)
Peptic ulcer disease	48 (2.1)	23 (2.2)
Schizophrenia	47 (2.1)	13 (1.2)
Irritable bowel syndrome	41 (1.8)	22 (2.1)
Multiple sclerosis	41 (1.8)	17 (1.6)
Parkinson's disease	26 (1.2)	22 (2.1)
Chronic viral hepatitis B	24 (1.1)	10 (0.9)
Number of comorbidities, *n* (%) Median (IQR)	4 (3–6)	5 (3–6)
0	26 (1.2)	4 (0.4)
1	168 (7.5)	37 (3.5)
2–4	1043 (46.4)	454 (42.7)
5–7	788 (35.1)	437 (41.1)
≥8	223 (9.9)	131 (12.3)
Previous kidney transplant, *n* (%)	174 (7.7)	45 (4.2)
Coronary artery bypass graft, *n* (%)	166 (7.4)	111 (10.4)
Percutaneous coronary intervention, *n* (%)	254 (11.3)	162 (15.2)
Dialysis vintage (years)		
Mean (SD)	3.5 (3.3)	3.6 (3.2)
Median (IQR)	2.4 (1.1–4.7)	2.6 (1.2–4.9)

SD: standard deviation; TIA: transient ischaemic attack.

Baseline characteristics assessed on 31 March 2019.

### Medication use

**Figure 1:**
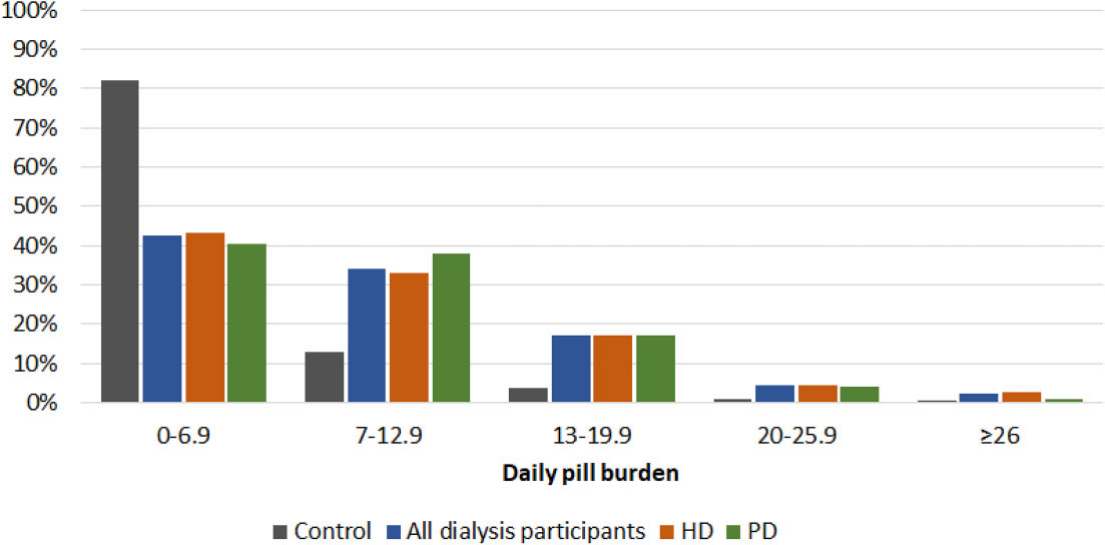
Distribution of pill burden by dialysis modality. Based on the 29 drug categories during the 120-day period prior to 31 March 2019 (controls: *n* = 22 480; all dialysis participants: *n* = 2248; HD: *n* = 1781; PD: *n* = 467).

Dialysis participants were prescribed a median of 6 (IQR 4–8) drug categories when considering medications dispensed in the 120-day period on or prior to 31 March 2019, corresponding to a median daily pill burden of 8.0 (IQR 4.6–12.6; range 0.02–52.4). A substantial number of dialysis participants were prescribed a very high number of pills per day (Fig. 1), with 5% having a pill burden of ≥21.7 pills/day and 16.5% taking ≥15 pills/day. There was no difference in pill burden between those receiving HD and PD [median 7.9 (IQR 4.4–12.7) versus 8.4 (IQR 5.2–12.4) pills/day, respectively; *P* = .52]. Control participants had a median daily pill burden of 2.3 (IQR 0.9–5.4), which was markedly lower than in participants on HD and PD (Table 2).

**Table 2:** Drug use characteristics based on the 29 drug categories of interest.

Characteristics	Dialysis participants (*n* = 2248	HD (*n* = 1781)	PD (*n* = 467)	*P*-value^a^	Controls without kidney failure (*n* = 22 480)
Number of unique drug categories					
Median (IQR)	6 (4–8)	6 (4–8)	6 (4–8)	.28	3 (1–4)
0	0	0	0	–	1188 (5.3)
1–4	742 (33.0)	599 (33.6)	143 (30.6)	.28	15 829 (70.4)
5–8	1132 (50.4)	879 (49.4)	253 (54.2)		4878 (21.7)
9–12	349 (15.5)	284 (16.0)	65 (13.9)		566 (2.5)
13–16	25 (1.1)	19 (1.1)	6 (1.3)		19 (0.1)
≥17	0	0	0		0 (0)
Daily pill burden, median (IQR)	8.0 (4.6–12.6)	7.9 (4.4–12.7)	8.4 (5.2–12.4)	.52	2.3 (0.9–5.4)
Number of prescriber specialties^b^					
Median (IQR)	2 (1–2)	2 (1–2), range 1–6	2 (1–2), range 1–5	<.001	1 (1–1)
1	859 (38.9)	712 (40.6)	147 (32.3)	.002	16 790 (84.5)
2	938 (42.5)	734 (41.9)	204 (44.8)		2677 (13.5)
≥3	411 (18.6)	307 (17.5)	104 (22.9)		396 (2.0)
Prescribing physician specialty^c^					
Nephrologist	1180 (52.5)	940 (52.8)	240 (51.4)	<.001	52 (0.2)
GP	589 (26.2)	500 (28.1)	89 (19.1)		16 649 (74.1)
Missing prescriber information	323 (14.4)	235 (13.2)	88 (18.8)		2822 (12.6)
Other	68 (3.0)	53 (3.0)	15 (3.2)		1005 (4.5)
Internist	62 (2.8)	36 (2.0)	26 (5.6)		384 (1.7)
Ophthalmologist	16 (0.7)	12 (0.7)	4 (0.9)		287 (1.3)
Cardiologist	10 (0.4)	5 (0.3)	5 (1.1)		93 (0.4)
Not applicable	0	0	0	–	1188 (5.3)
**Based on PIM category in patients ≥65 years of age**	**Dialysis participants (*n* = 1063)**	**HD (*n* = 866)**	**PD (*n* = 197)**	***P*-value^a^**	**Controls without kidney failure (*n* = 10 599)**
Number of unique drug categories, median (IQR)	2 (1–2)	2 (1–2)	1 (1–2)	.042	1 (0–1)
Daily pill burden, median (IQR)	1.2 (0.5–2.4)	1.2 (0.6–2.5)	1.1 (0.2–2.2)	.073	0.3 (0–1.1)

Characteristics are based on medication use during the 120-day period prior to 31 March 2019.

SD: standard deviation.

a Difference between HD and PD.

b For *n* = 40 in the dialysis cohort and *n* = 1429 in the control cohort without kidney failure, all prescriptions dispensed had missing prescriber information and were excluded. A further 1188 in the control cohort with no prescriptions of interest in the period were also excluded.

c Based on the single prescription closest to 31 March 2019.

After the ‘miscellaneous’ drug category, the 10 most commonly prescribed drug categories in descending order (HD and PD combined) were statins, PPIs, beta blockers, calcium channel blockers, vitamin D or vitamin D analogues, diuretics, insulin, opioids, benzodiazepines/hypnotics and prescription aspirin (Supplementary Fig. S2).

Prescriber information was missing for ≈14% of prescriptions when considering the single most recent in the 120-day period on or prior to 31 March 2019. Nephrologists were the most common prescriber (52.5%), followed by primary care practitioners (26.2%), among all participants receiving maintenance dialysis. General practitioners were the most common prescriber (74.1%) in control participants (Table 2).

**Figure 2:**
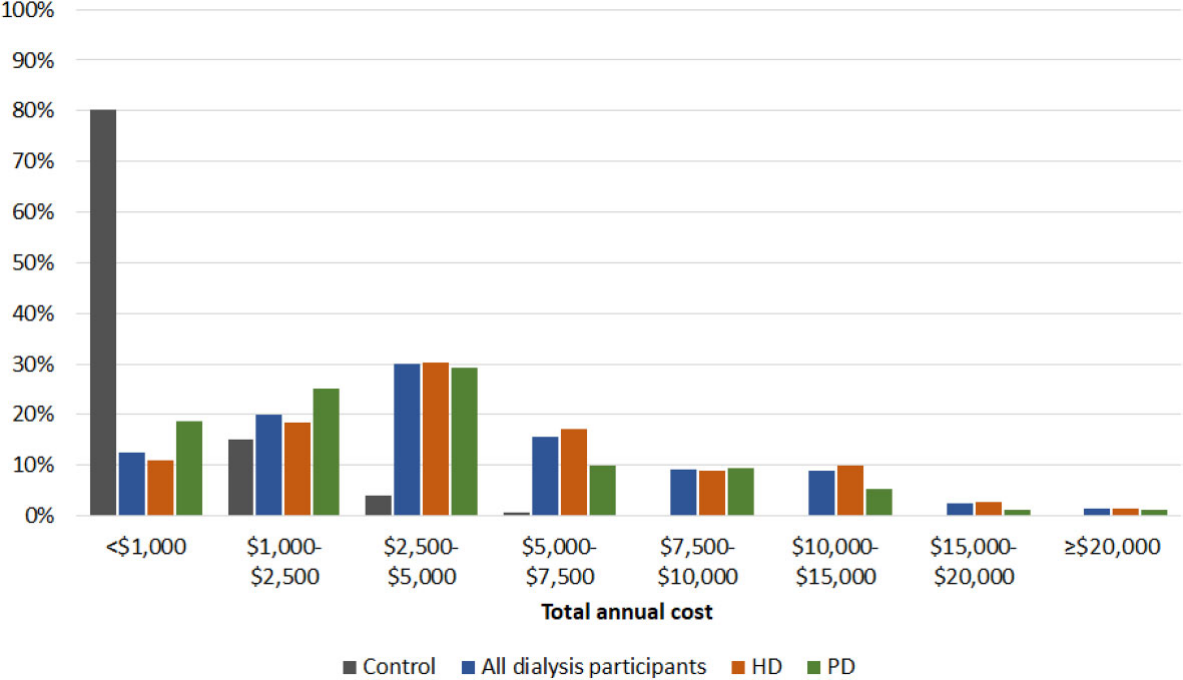
Distribution of total annual medication costs by dialysis modality (in 2021 Canadian dollars). Based on the 29 drug categories of interest during the 1-year period prior to 31 March 2019 (controls: n = 22 480; all dialysis participants: n = 2248; HD: n = 1781; PD: n = 467). For example, when considering all dialysis participants, ≈20% have total annual medication costs between ${\$}$1000 and ${\$}$2500.

The median annual per-participant cost for all medications was ${\$}$3831, or ≈${\$}$11.6 million annually for all dialysis participants (HD or PD). The median annual cost per participant was higher for participants treated with HD (${\$}$4087) compared with PD (${\$}$2982). As with pill burden, the annual medication cost was right-skewed, with some participants having very high annual medication costs (Fig. 2). For the 22 480 control participants, the median annual cost per participant was ${\$}$340.

Ranking by annual cost, the top 10 drug categories among dialysis participants (including oral hypoglycaemics) accounted for the great majority (95.7%) of total costs: median annual per-participant cost of ${\$}$3585, or ≈${\$}$11.1 million annually for all participants. Among the top 10 drug categories, the ‘miscellaneous’ drug category accounted for ≈83% of the total annual cost (Table 3), driven by high costs associated with erythropoietin stimulating agents (ESAs).

**Table 3:** Top 10 drug categories, ranked by annual cost (in Canadian dollars).

Medication	Annual cost	Number of participants with at least one prescription in the 1 year on or prior to 31 March 2019	Mean annual cost per participant (based on all participants)	Median cost per participant (based on all participants)
All dialysis participants (*N* = 2248)				
Miscellaneous	9 179 782	2207	4084	2812
Darbepoetin/erythropoietin	7 186 898	1729	3197	2144
Other	1 992 884	2092	887	252
Insulin	520 796	784	232	0
Vitamin D or vitamin D analogues	213 111	1007	95	0
Diuretics	207 591	871	92	0
Beta blockers	196 651	1184	87	18
Opioids	190 506	1026	85	0
Calcium channel blockers	168 362	1107	75	0
PPIs	159 635	1209	71	26
Statins	119 309	1230	53	37
Oral hypoglycaemics	116 537	309	52	0
HD participants (*n* = 1781)				
Miscellaneous	7 749 587	1753	4351	2989
Darbepoetin/erythropoietin	6 150 178	1421	3453	2360
Other	1 599 408	1646	898	254
Insulin	399 278	614	224	0
Vitamin D or vitamin D analogues	187 065	827	105	0
Opioids	173 602	834	97	0
Beta blockers	155 603	945	87	21
PPIs	131 905	987	74	35
Diuretics	131 034	600	74	0
Calcium channel blockers	120 076	828	67	0
Statins	92 186	948	52	28
Oral hypoglycaemics	88 569	244	50	0
PD participants (*n* = 467)				
Miscellaneous	1 430 196	454	3063	1685
Darbepoetin/erythropoietin	1 036 720	308	2220	1133
Other	393 476	446	843	245
Insulin	121 517	170	260	0
Diuretics	76 556	271	164	79
Calcium channel blockers	48 286	279	103	45
Beta blockers	41 045	239	88	10
Oral hypoglycaemics	27 967	65	60	0
PPIs	27 730	222	59	0
Statins	27 124	282	58	51
Vitamin D or vitamin D analogues	26 046	180	56	0
Angiotensin receptor blockers	17 357	139	37	0
Controls without kidney failure (*n* = 22 480)				
Miscellaneous	4 703 991	17 633	209	51
Darbepoetin/erythropoietin	113 304	21	5	0
Other	4 590 687	17 631	204	51
Oral hypoglycaemics	1 759 580	3490	78	0
Opioid	1 082 958	5448	48	0
Antidepressants	1 070 030	5036	48	0
Insulin	931 279	1043	41	0
PPIs	830 739	6435	37	0
Statins	749 569	7923	33	0
Angiotensin-converting enzyme inhibitors	593 870	5028	26	0
Calcium channel blockers	562 775	3827	25	0
Beta blockers	531 150	4014	24	0

Annual cost is assessed based on prescriptions in the 1 year on or prior to 31 March 2019. Mean and median annual cost is calculated based on all participants in the cohort (regardless of whether they had a prescription for the study medication of interest). For example, in the *n* = 2248 dialysis cohort, although there were 784 participants who used insulin in the period of interest, the mean annual cost based on all 2248 participants was ${\$}$232; in the *n* = 1781 PD cohort, although there were 614 participants who used insulin, the mean annual cost based on all 1781 participants was ${\$}$224.

The top 10 drug categories ranked by annual cost differ when examining all 2248 dialysis patients compared with HD only, PD only and controls.

Not all drugs were covered by Alberta Health; these drugs accounted for <3% of the quantity dispensed within each study drug category with the exception of antidepressants, fibrates, opioids, ‘miscellaneous’, prescription NSAIDs other than aspirin and prescription aspirin (3.1%, 7.4%, 8.5%, 12.6%, 19.9% and 94.1%, respectively, of the quantity dispensed was not covered by Alberta Health ) for the dialysis cohort.

‘Miscellaneous’, PPIs, fibrates, prescription NSAIDs other than aspirin, opioids, vitamin D or vitamin D analogues and prescription aspirin (4.5%, 7.2%, 10.7%, 14.9%, 19.4%, 50.1% and 91.7%, respectively) for the control cohort without kidney failure.

Costs were imputed for drugs with missing costs based on the median unit price for the corresponding drug within each drug category with the exception of prescription aspirin, which was excluded due to most costs missing. For drugs that had no costs for which to impute, other provincial drug formularies were searched to assign unit prices.

### Use of contraindicated medications

Less than 1% of dialysis participants were taking each of anticoagulants, anticonvulsants, antihyperlipidaemics, antineoplastics, anti-Parkinson’s, bisphosphonates, dementia medications and ‘other’ medications in the 120-day period on or before 31 March 2019. A total of 1.3%, 1.4%, 3.7% and 4.7% of participants were taking hypoglycaemics, psychotherapeutic agents, anti-infectives and NSAIDs (excluding aspirin), respectively (Supplementary Table S4). HD participants were more often prescribed NSAIDs (other than aspirin) compared with PD participants (5.3% versus 2.6%; *P* = .01).

### Use of PIMs

Among dialysis participants ≥65 years of age, the median number of PIM categories dispensed in the 120-day period on or prior to 31 March 2019 was 2 (IQR 1–2) (Table 2). The corresponding median daily pill burden associated with these PIMs was 1.2 (IQR 0.5–2.4). The median number of PIM categories for participants treated with HD was higher than for participants treated with PD [2 (IQR 1–2) versus 1 (1–2); *P* = .04] (Table 2). The top five most commonly prescribed PIMs were PPIs, NSAIDs, insulin, first-generation antihistamines and benzodiazepines. Participants treated with HD were prescribed more peripheral alpha-1 blockers and NSAIDs than those treated with PD (*P* = 0.01 and 0.03, respectively), while the reverse was observed for hormones (*P* = .02) (Supplementary Table S5).

## DISCUSSION

In this analysis of 2248 participants on dialysis, we found that participants treated with maintenance HD or PD were prescribed a median of six separate drug categories and two PIM categories. Nearly 17% of all participants on dialysis were prescribed nine or more drug categories, 16.5% were prescribed ≥15 pills/day and 12% were prescribed at least one medication that is contraindicated in kidney failure. The median cost per participant was ${\$}$3831/year, with 13% having annual costs exceeding ${\$}$10 000. Pill burden and associated costs for participants on maintenance dialysis were >3-fold and 10-fold higher, respectively, as compared with age- and sex-matched controls without kidney failure.

Despite the similar rates of prescribed medications and daily pill burden, participants on HD in our study tended to have a much higher annual cost per participant (median ${\$}$4087) versus participants on PD (median ${\$}$2982), driven in part by greater use of ESAs [32–34]. More proactive use of intravenous iron may reduce ESA requirements [35] and thereby reduce medication-related costs for patients on HD. In our centre, intravenous iron is administered during routine dialysis sessions, reducing the additional costs (e.g. personnel, facility space) that are associated with this treatment.

Like previous work, our study shows that participants on dialysis are commonly prescribed statins, PPIs, beta blockers, calcium channel blockers, vitamin D or vitamin D analogues, diuretics and insulin [4, 7, 10, 11, 20] and are prescribed a median of two PIM categories, with the most common being PPIs and NSAIDs. We found that ≈38% of participants with diabetes in our cohort were not prescribed insulin or oral antihyperglycaemics. Previous studies of patients with diabetes and treated with dialysis have shown that 23% are not treated with pharmacotherapy and ≈30% have a haemoglobin A1c <6%, likely due to changes in insulin metabolism that occur in kidney failure [36, 37]. Our study adds to existing knowledge by highlighting differences in medication prescriptions and costs between participants on HD and PD and between participants on dialysis and those in the general population. Further, we also provide data on the use of contraindicated medications in participants with kidney failure. The use of such medications in our cohort was relatively low. Among the medications that we considered as contraindicated, NSAIDs were the most prescribed medication in this cohort (4.7%) and were more commonly used in participants on HD versus PD, perhaps because preserving residual kidney function is a higher priority in the latter [38]. Recent data suggest that NSAIDs may be safer in patients with chronic kidney disease (CKD) than opioids despite their nephrotoxic effects [39] and their benefits may outweigh the harms in selected patients. Our data suggest the need for further study of other contraindicated medications that focus on the risk:benefit ratio and to consider patient values and preferences. Further work is also needed to define a list of PIMs that is specifically applicable for use in patients on dialysis. For example, insulin is one of a limited number of antihyperglycaemics that is safe for use in patients on dialysis [40] and thus it likely does not represent a PIM in dialysis populations.

Participants in our study were prescribed fewer medications than reported in other dialysis populations, which typically reported >10 medications [5, 7, 11], and participants in our study also tended to have fewer prescribers. In a recent Canadian study of patients on HD, the median number of prescribers was 3 and 54% of patients had ≥3 prescribers, as compared to a median of 2 in our study, with only 19% of patients having ≥3 prescribers [4]. Patients in this study were also older and had higher dialysis vintage compared with our cohort. Unlike prior work, where general practitioners (GPs) or family physicians were the most common prescribers (49.4%) [4], nephrologists were the main prescribers (53%) to the participants in our study. Further, the use of contraindicated medications in our cohort was lower than in previous reports of CKD patients managed in the primary care setting [41]. Whether this is due to specific practices in our dialysis program (such as regular medication reviews or support from specialized renal pharmacists) [13, 42–44] would require further study. Further work is needed to compare prescribing patterns between centres with and without specialized renal pharmacists. One might also speculate that GPs may be reluctant to deprescribe medications in patients on dialysis, especially if they were started by specialists [45].

Data on the subset of patients on dialysis who are prescribed a very large number of medications is lacking. The term ‘hyperpolypharmacy’ has been used to define the extremes of polypharmacy and refers to the use of ≥10 medications [46]. However, this threshold does not have much discriminative power in patients on dialysis, given that the median number of prescribed medications is typically ≥10 [4, 7, 11, 20]. Future work is needed to define new thresholds for ‘polypharmacy’ and ‘hyperpolypharmacy’ among patients receiving maintenance HD and PD.

Our study has several strengths, including providing a comprehensive analysis of the patterns and costs relating to prescription medications in a community cohort of participants on HD and PD, assessment of PIMs and contraindicated medications and comparison to a cohort without kidney failure. Nonetheless, our study has several limitations that are worth noting. First, we could not include all over-the-counter (OTC) medications, since some but not all OTC medications are included in the provincial PIN database. Previous analysis that included OTC medications showed that participants on HD are prescribed a greater number of total prescriptions, PIMS and as needed medications versus those on PD [11]. Second, certain specialized medications (e.g. recombinant tissue plasminogen activator) are dispensed by the dialysis units and are not captured in PIN data. These two considerations may partially account for the lower number of medications for the participants in our study as compared with prior work. Third, our list of medications that are contraindicated in kidney failure is not a comprehensive list but is meant to represent a concise list of commonly prescribed medications [26–28]. Similarly, some medications that were listed as contraindicated can likely be safely used in patients with kidney failure—including NSAIDs, but also fibrates [47–49]. Fourth, our control cohort was not matched for comorbidity status with the dialysis cohort and therefore the differences in prescription patterns between the two groups likely relate to comorbidity burden as well as the presence of kidney failure per se [50]. Fifth, we assessed annual medication costs for all participants during a look-back period of 1 year but classified receipt of dialysis and dialysis modality at baseline. The advantage of this approach is that it increased the sample size and improved generalizability by reducing the likelihood of survivorship bias. The potential disadvantage is that some participants will not have received dialysis during the entire look-back period and a smaller number will have been misclassified with respect to dialysis modality. To the extent that initiating dialysis was more common (14.8%) than modality switches (6.6%), and because medication use typically increases following dialysis initiation, this approach will have tended to underestimate true annual costs among patients receiving dialysis. Sixth, some of the prescriptions in our cohort reflect local protocols that may differ from dialysis programs in other countries. This may partially explain differences in prescribing patterns between our study and others, but may also limit the generalizability of some of our findings. Finally, our study was unable to assess long-term adherence to the prescribed medications.

In conclusion, our study of participants receiving HD or PD found a median number of prescribed medications that was lower than other prior studies but still markedly higher than the general population. Participants on dialysis were prescribed a median of 2 PIM categories and had a relatively low but non-negligible use of contraindicated medications. These findings validate the potential value of regular medication review for dialysis patients and support the need for new interventions that safely reduce polypharmacy in this population.

## Supplementary Material

gfae154_Supplemental_File

## Data Availability

We cannot make our dataset available to other researchers due to our contractual arrangements with the provincial health ministry (Alberta Health), who is the data custodian. Researchers may make requests to obtain a similar dataset at https://sporresources.researchalberta.ca.
