# Supplementary material for: Prescribing patterns and medication costs in patients on maintenance haemodialysis and peritoneal dialysis
Source: Nephrol Dial Transplant. 2024 Jul 4;40(2):360–70. doi: 10.1093/ndt/gfae154 (PMC11852291; doi:10.1093/ndt/gfae154)
Supplement: gfae154_Supplemental_File [file gfae154_Supplemental_File.docx]

**Supplemental Figure 1: Flow diagram**

AKDN Alberta Kidney Disease Network; HD hemodialysis; PD peritoneal dialysis

**Supplemental Figure 2: Percentage of dialysis participants receiving at least one medication by each drug category**


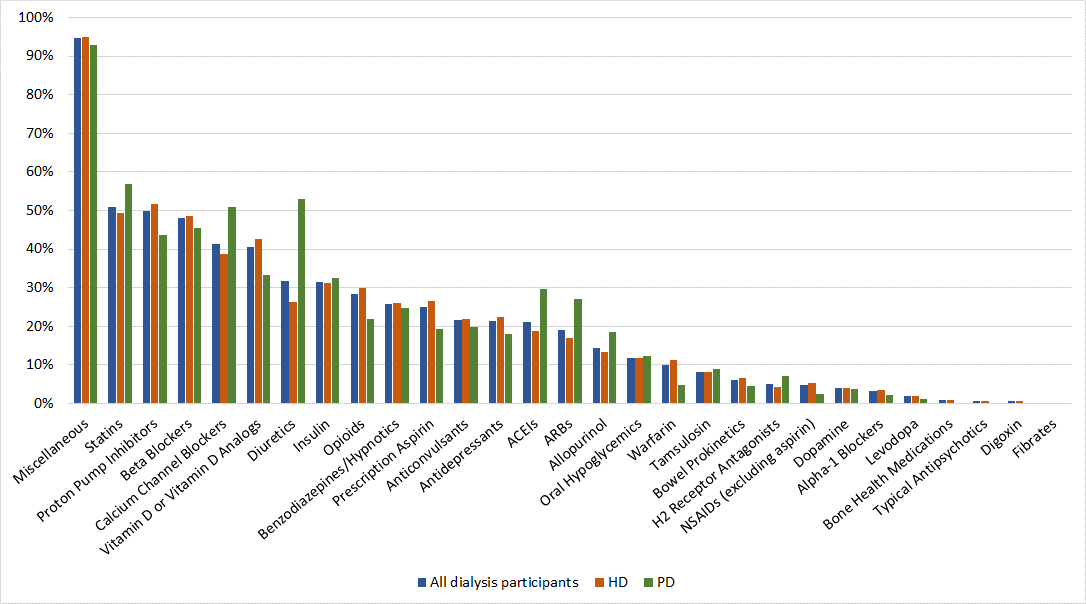


ACEI angiotensin-converting enzyme inhibitors; ARB angiotensin receptor blockers; HD hemodialysis; NSAIDS nonsteroidal anti-inflammatory drugs; PD peritoneal dialysis

Percentage is based on evidence of at least 1 prescription within the drug category in the 120 day period on or prior to March 31, 2019. For example, approximately 50% of the N=2,248 dialysis participants had at least 1 prescription for statins. Approximately 20% had at least 1 prescription for ACEIs. Sorted in descending order based on medications taken by the N=2,248 dialysis participants. (All dialysis: N=2,248; HD: N=1,781; PD: N=467)

**Supplemental Table 1: Study Drug List**

| Drug Category | Ingredients |
| --- | --- |
| Allopurinol | ALLOPURINOL |
| Alpha-1 Blockers | DOXAZOSIN, PRAZOSIN, TERAZOSIN |
| Angiotensin-Converting Enzyme Inhibitors (ACEI) | BENAZEPRIL, CAPTOPRIL, CILAZAPRIL, ENALAPRIL, FOSINOPRIL, LISINOPRIL, PERINDOPRIL, QUINAPRIL, RAMIPRIL, TRANDOLAPRIL |
| Angiotensin Receptor Blockers (ARB) | AZILSARTAN, CANDESARTAN, EPROSARTAN, IRBESARTAN, LOSARTAN, OLMESARTAN, TELMISARTAN, VALSARTAN |
| Anticonvulsants | GABAPENTIN, PREGABALIN |
| Antidepressants | AMITRIPTYLINE, AMOXAPINE, BUPROPION, CITALOPRAM, CLOMIPRAMINE, DESIPRAMINE, DESVENLAFAXINE, DOXEPIN, DULOXETINE, ESCITALOPRAM, FLUOXETINE, FLUVOXAMINE, IMIPRAMINE, ISOCARBOXAZID, LEVOMILNACIPRAN, MAPROTILINE, MIRTAZAPINE, MOCLOBEMIDE, NEFAZODONE, NORTRIPTYLINE, PAROXETINE, PHENELZINE, PROTRIPTYLINE, SELEGILINE, SERTRALINE, TRANYLCYPROMINE, TRAZODONE, TRIMIPRAMINE, VENLAFAXINE, VILAZODONE, VORTIOXETINE |
| Typical Antipsychotics | CHLORMEZANONE, CHLORPROMAZINE, CHLORPROTHIXENE, FLUPENTIXOL, FLUPHENAZINE, FLUSPIRILENE, HALOPERIDOL, LOXAPINE, MESORIDAZINE, METHOTRIMEPRAZINE, PERICIAZINE, PERPHENAZINE, PIMOZIDE, PIPOTIAZINE, PROCHLORPERAZINE, THIOPROPAZATE, THIOPROPERAZINE, THIORIDAZINE, THIOTHIXENE, TRIFLUOPERAZINE, ZUCLOPENTHIXOL |
| Prescription Aspirin | ACETYLSALICYLIC ACID, ACETYLSALICYLIC ACID & BUTALBITAL & CAFFEINE, ACETYLSALICYLIC ACID & BUTALBITAL & CAFFEINE & CODEINE PHOSPHATE, ACETYLSALICYLIC ACID & CAFFEINE, ACETYLSALICYLIC ACID & CAFFEINE & CODEINE PHOSPHATE, ACETYLSALICYLIC ACID & CAFFEINE CITRATE & CODEINE PHOSPHATE, ACETYLSALICYLIC ACID & CAFFEINE & CODEINE PHOSPHATE & MEPROBAMATE, ACETYLSALICYLIC ACID & CAFFEINE & DEXTROPROPOXYPHENE HCL, ACETYLSALICYLIC ACID & CALCIUM CARBONATE & MAGNESIUM OXIDE & MAGNESIUM CARBONATE, ACETYLSALICYLIC ACID & CHLORPHENIRAMINE MALEATE, ACETYLSALICYLIC ACID & CODEINE PHOSPHATE & MAGNESIUM HYDROXIDE & ALUMINUM HYDROXIDE, ACETYLSALICYLIC ACID & DIPYRIDAMOLE, ACETYLSALICYLIC ACID & OXYCODONE HYDROCHLORIDE, ACETYLSALICYLIC ACID & METHOCARBAMOL, ACETYLSALICYLIC ACID & METHOCARBAMOL & CODEINE PHOSPHATE, ACETYLSALICYLIC ACID & NIFEDIPINE, ACETYLSALICYLIC ACID & SODIUM BICARBONATE & CITRIC ACID |
| Benzodiazepines/Hypnotics | ALPRAZOLAM, BROMAZEPAM, CHLORDIAZEPOXIDE, CLOBAZAM, CLONAZEPAM, CLORAZEPATE, DIAZEPAM, ESTAZOLAM, FLUMAZENIL, FLURAZEPAM, KETAZOLAM, LORAZEPAM, MELATONIN, MIDAZOLAM, NITRAZEPAM, OXAZEPAM, TEMAZEPAM, TRIAZOLAM, ZOLPIDEM, ZOPICLONE |
| Beta Blockers | ACEBUTOLOL, ATENOLOL, BETAXOLOL, BISOPROLOL, BRIMONIDINE, CARVEDILOL, ESMOLOL, LABETALOL, LEVOBUNOLOL, METOPROLOL, NADOLOL, NEBIVOLOL, OXPRENOLOL, PINDOLOL, PROPRANOLOL, SOTALOL, TIMOLOL |
| Bone Health Medications^a^ | ALENDRONATE, CALCITONIN, CLODRONATE, CLODRONIC, DENOSUMAB, ETIDRONATE, IBANDRONATE, PAMIDRONATE, RALOXIFENE, RISEDRONATE, ROMOSOZUMAB, TERIPARATIDE, ZOLEDRONATE |
| Bowel Prokinetics | CISAPRIDE, DOMPERIDONE, METOCLOPRAMIDE |
| Calcium Channel Blockers | AMLODIPINE, CLEVIDIPINE, DILTIAZEM, ERYTHRITYL, FELODIPINE, NICARDIPINE, NIFEDIPINE, NIMODIPINE, VERAPAMIL |
| Digoxin | DIGOXIN |
| Diuretics | ACETAZOLAMIDE, AMILORIDE, BENDROFLUMETHIAZIDE, BUMETANIDE, CHLORTHALIDONE, EPLERENONE, ETHACRYNIC, FUROSEMIDE, HYDROCHLOROTHIAZIDE, INDAPAMIDE, METHYCLOTHIAZIDE, METOLAZONE, SPIRONOLACTONE, TORSEMIDE, TRIAMTERENE |
| Dopamine Agonists | APOMORPHINE, PRAMIPEXOLE, ROPINIROLE, ROTIGOTINE |
| Fibrates | BEZAFIBRATE, CLOFIBRATE, FENOFIBRATE, GEMFIBROZIL |
| H2 Receptor Antagonists | CIMETIDINE, FAMOTIDINE, NIZATIDINE, RANITIDINE |
| Oral Hypoglycemics | ACARBOSE, ACETOHEXAMIDE, ALOGLIPTIN, CANAGLIFLOZIN, CHLORPROPAMIDE, DAPAGLIFLOZIN, EMPAGLIFLOZIN, ERTUGLIFLOZIN, GLICLAZIDE, GLIMEPIRIDE, GLYBURIDE, LINAGLIPTIN, METFORMIN, NATEGLINIDE, PIOGLITAZONE, REPAGLINIDE, ROSIGLITAZONE, SAXAGLIPTIN, SITAGLIPTIN, TOLBUTAMIDE |
| Insulin | INSULIN |
| Levodopa | LEVODOPA |
| Prescription Nonsteroidal Anti-Inflammatory Drugs (NSAIDs) other than aspirin | BENZYDAMINE, CELECOXIB, DICLOFENAC, DIFLUNISAL, ETODOLAC, FENOPROFEN, FLOCTAFENINE, FLURBIPROFEN, IBUPROFEN, INDOMETHACIN, KETOPROFEN, KETOROLAC, MEFENAMIC, MELOXICAM, NABUMETONE, NAPROXEN, OXAPROZIN, OXYPHENBUTAZONE, PHENYLBUTAZONE, PIROXICAM, ROFECOXIB, SULINDAC, TENOXICAM, TIAPROFENIC, TOLMETIN, VALDECOXIB |
| Opioids | ALFENTANIL, ANILERIDINE, BUPRENORPHINE, BUTORPHANOL, CODEINE, DEXTROPROPOXYPHENE, FENTANYL, HYDROCODONE, HYDROMORPHONE, LEVORPHANOL, MEPERIDINE, METHADONE, MORPHINE, NALBUPHINE, NALOXONE, NALTREXONE, OPIUM, OXYCODONE, OXYMORPHONE, PENTAZOCINE, PROPOXYPHENE, REMIFENTANIL, SUFENTANIL, TAPENTADOL, TRAMADOL |
| Proton Pump Inhibitors | DEXLANSOPRAZOLE, ESOMEPRAZOLE, LANSOPRAZOLE, OMEPRAZOLE, PANTOPRAZOLE, RABEPRAZOLE |
| Statins | ATORVASTATIN, CERIVASTATIN, FLUVASTATIN, LOVASTATIN, PRAVASTATIN, ROSUVASTATIN, SIMVASTATIN |
| Tamsulosin | TAMSULOSIN |
| Vitamin D or Vitamin D Analogs^b^ | ALFACALCIDOL, CALCIFEDIOL, CALCITRIOL, DIHYDROTACHYSTEROL, DOXERCALCIFEROL, PARICALCITOL, VITAMIN D, VITAMIN D2, VITAMIN D3 |
| Warfarin | WARFARIN |
| Miscellaneous  Drug classes that were used by more than 10 participants within 120 days on or prior to the index date (and not previously flagged in the 28 other categories)^c^ | ACETAMINOPHEN, AMIODARONE, AMOXICILLIN, AMOXICILLIN AND BETA-LACTAMASE INHIBITOR, AZATHIOPRINE, AZITHROMYCIN, BECLOMETHASONE, BETAHISTINE, BETAMETHASONE, BIMATOPROST, BISACODYL, BROMFENAC, BUDESONIDE, CEFALEXIN, CEFIXIME, CEFUROXIME, CETIRIZINE, CICLOSPORIN, CINACALCET, CIPROFLOXACIN, CLARITHROMYCIN, CLINDAMYCIN, CLOBETASOL, CLONIDINE, CLOPIDOGREL, CLOTRIMAZOLE, CLOXACILLIN, COLCHICINE, CYCLOBENZAPRINE, DARBEPOETIN ALFA, DEXAMETHASONE, DIPHENHYDRAMINE, DOCUSATE SODIUM, DOXYCYCLINE, DUTASTERIDE, EPINEPHRINE, ERYTHROPOIETIN, EZETIMIBE, FEBUXOSTAT, FINASTERIDE, FLUCONAZOLE, FLUTICASONE, FOLIC ACID, FORMOTEROL AND BUDESONIDE, FUSIDIC ACID, GATIFLOXACIN, GLUCAGON, GLUCOSAMINE & CHONDROITIN^d^, GLYCERYL TRINITRATE, HYDRALAZINE, HYDROCORTISONE, HYDROXYCHLOROQUINE, HYDROXYZINE, IMIDAZOLES/TRIAZOLES IN COMBINATION WITH CORTICOSTEROIDS, ISOSORBIDE MONONITRATE, LACTULOSE, LAMOTRIGINE, LATANOPROST, LEVETIRACETAM, LEVOFLOXACIN, LEVOTHYROXINE SODIUM, LIDOCAINE, LOPERAMIDE, MACROGOL, MAGNESIUM GLUCONATE, METRONIDAZOLE, MIDODRINE, MINOXIDIL, MOMETASONE, MONTELUKAST, MOXIFLOXACIN, MULTIENZYMES, MUPIROCIN, MYCOPHENOLIC ACID, NYSTATIN, OLANZAPINE, ONDANSETRON, PHENYTOIN, POLYSTYRENE SULPHONATE, POTASSIUM CHLORIDE, PREDNISOLONE, PREDNISONE, QUETIAPINE, QUININE, RISPERIDONE, SALBUTAMOL, SALMETEROL AND FLUTICASONE, SEVELAMER, SILDENAFIL, SULFAMETHOXAZOLE AND TRIMETHOPRIM, TACROLIMUS, TADALAFIL, TERBINAFINE, TETRAHYDROCANNABINOL^d^, TICAGRELOR, TINZAPARIN, TOBRAMYCIN, TRAVOPROST, TRIAMCINOLONE, UMECLIDINIUM BROMIDE, VALACICLOVIR, VALPROIC ACID, VANCOMYCIN, VARENICLINE, VILANTEROL AND FLUTICASONE FUROATE, VITAMIN B-COMPLEX WITH VITAMIN C |

The drug lists are based on Supplemental Table 2 from “A Province-Wide, cross-sectional study of demographics and medication use of patients in hemodialysis units across Ontario” by Battistella et al.^4^ and lists from Alberta Health and searching the Health Canada Drug Database. The lists are comprised of drugs that were searched for but did not necessarily exist in the cohort due to the following reasons: (the drug no longer being prescribed during the period of interest, the drug not yet available during the period of interest, the drug having never been available to prescribe in Canada, or just not prescribed for any participant in the cohort during the period of interest).

^a^The drug category “bone health medications” was originally called “bisphosphonates” in Battistella et al. We expanded the class and renamed accordingly.

^b^The drug category “Vitamin D and Vitamin D analogs” was originally called “calcitriol” in Battistella et al. We expanded the class and renamed accordingly.

^c^Drug classes in the miscellaneous category are based on the Anatomical Therapeutic Chemical (ATC) classification system as per the Health Canada Drug Product Database. If more than one ATC code was assigned to a particular drug identification number, the first code was used.

**^d^** These drugs were originally classified as NSAIDs in Battistella et al.; we reclassified them in the miscellaneous category

**Supplemental Table 2: Contraindicated medications in kidney failure**

| Drug Category | Ingredients |
| --- | --- |
| Anticoagulants | APIXABAN, DABIGATRAN, EDOXABAN, FONDAPARINUX, RIVAROXABAN |
| Anticonvulsants | ACETAZOLAMIDE |
| Antihyperlipidemics | FENOFIBRATE, GEMFIBROZIL |
| Antiinfectives | TRIMETHOPRIM, NITROFURANTOIN |
| Antineoplastics | METHOTRAXATE |
| Anti Parkinsons | SELEGILINE |
| Bisphosphonates | ALENDRONATE, IBANDRONATE, RISEDRONATE, ZOLEDRONIC ACID |
| Dementia | GALANTAMINE |
| Hypoglycemics | ACARBOSE, CANAGLIFLOZIN, DAPAGLIFLOZIN, EMPAGLIFLOZIN, GLYBURIDE, METFORMIN, SAXAGLIPTIN |
| NSAIDs (other than aspirin) | See list from **Supplemental Table 1** |
| Psychotherapeutic Agents | BUSPIRONE, DULOXETINE |
| Other | BACLOFEN, TOLTERODINE |

NSAIDs Nonsteroidal Anti-Inflammatory Drugs

**Supplemental Table 3: Potentially inappropriate medications in older adults**

Based on Table 2 and Table 3 from American Geriatrics Society 2015 Updated Beers Criteria for Potentially Inappropriate Medication Use in Older Adults^1^

| Drug Category | Ingredients |
| --- | --- |
| Central Alpha Blockers | CLONIDINE, GUANABENZ, GUANFACINE, METHYLDOPA, RESERPINE |
| Peripheral Alpha-1 Blockers | DOXAZOSIN, PRAZOSIN, TERAZOSIN |
| Androgens | METHYLTESTOSTERONE, TESTOSTERONE |
| Antiarrhythmics | AMIODARONE, DIGOXIN, DISOPYRAMIDE, DRONEDARONE |
| Antidepressants | AMITRIPTYLINE, AMOXAPINE, CLOMIPRAMINE, DESIPRAMINE, DOXEPIN, IMIPRAMINE, NORTRIPTYLINE, PAROXETINE, PROTRIPTYLINE, TRIMIPRAMINE |
| Antihistamines (1^st^ generation) | BROMPHENIRAMINE, CARBINOXAMINE, CHLORPHENIRAMINE, CLEMASTINE, CYPROHEPTADINE, DEXBROMPHENIRAMINE, DEXCHLORPHENIRAMINE, DIMENHYDRINATE, DIPHENHYDRAMINE (only oral), DOXYLAMINE, HYDROXYZINE, MECLIZINE, PROMETHAZINE, TRIPROLIDINE |
| Antiinfectives | NITROFURANTOIN |
| Anti Parkinsons | BENZTROPINE (only oral), TRIHEXYPHENIDYL |
| Antipsychotics (1^st^ and 2^nd^ generation) | ARIPIPRAZOLE, ASENAPINE, BREXPIPRAZOLE, CHLORMEZANONE, CHLORPROMAZINE, CHLORPROTHIXENE, CLOZAPINE, FLUPENTIXOL, FLUPHENAZINE, FLUSPIRILENE, HALOPERIDOL, LOXAPINE, LURASIDONE, MESORIDAZINE, METHOTRIMEPRAZINE, OLANZAPINE, PALIPERIDONE, PERICIAZINE, PERICYAZINE, PERPHENAZINE, PIMOZIDE, PIPOTIAZINE, PROCHLORPERAZINE, PROMAZINE, QUETIAPINE, RISPERIDONE, THIOPROPAZATE, THIOPROPERAZINE, THIORIDAZINE, THIOTHIXENE, TRIFLUOPERAZINE, ZIPRASIDONE, ZUCLOPENTHIXOL |
| Antispasmodics | ATROPINE (excludes ophthalmic), BELLADONNA, CHLORDIAZEPOXIDE, CLIDINIUM, DICYCLOMINE, HYOSCYAMINE, PROPANTHELINE, SCOPOLAMINE |
| Antithrombotics | DIPYRIDAMOLE, TICLOPIDINE |
| Barbiturates | AMOBARBITAL, BUTABARBITAL, BUTALBITAL, MEPHOBARBITAL, PENTOBARBITAL, PHENOBARBITAL, SECOBARBITAL |
| Benzodiazepines | ALPRAZOLAM, CHLORDIAZEPOXIDE, CLONAZEPAM, CLORAZEPATE, DIAZEPAM, ESTAZOLAM,  FLURAZEPAM, LORAZEPAM, OXAZEPAM, QUAZEPAM, TEMAZEPAM, TRIAZOLAM |
| Bowel Prokinetics | METOCLOPRAMIDE |
| Calcium Channel Blockers | NIFEDIPINE |
| Hormones | THYROID, DESMOPRESSIN, ESTROGEN, MEGESTROL, GROWTH HORMONE |
| Insulin | INSULIN |
| Laxatives | MINERAL OIL |
| Non-Benzodiazepine | ESZOPICLONE, ZALEPLON, ZOLPIDEM |
| NSAIDS (non-COX and COX-selective, oral and parenteral) | BENZYDAMINE, CELECOXIB, DICLOFENAC, DIFLUNISAL, ETODOLAC, FENOPROFEN, FLOCTAFENINE, FLURBIPROFEN, IBUPROFEN, INDOMETHACIN, KETOPROFEN, KETOROLAC, LUMIRACOXIB, MECLOFENAMATE, MEFENAMIC, MELOXICAM, NABUMETONE, NAPROXEN, OXAPROZIN, OXYPHENBUTAZONE, PHENYLBUTAZONE, PIROXICAM, ROFECOXIB, SULINDAC, TENOXICAM, TIAPROFENIC, TOLMETIN, VALDECOXIB |
| Opioids | MEPERIDINE, PENTAZOCINE |
| Proton Pump Inhibitors | DEXLANSOPRAZOLE, ESOMEPRAZOLE, LANSOPRAZOLE, OMEPRAZOLE, PANTOPRAZOLE, RABEPRAZOLE |
| Skeletal | CARISOPRODOL, CHLORZOXAZONE, CYCLOBENZAPRINE, METAXALONE, METHOCARBAMOL, ORPHENADRINE |
| Sulfonylureas | CHLORPROPAMIDE, GLYBURIDE |
| Sympatholytics | ERGOLOID |
| Tranquilizers | MEPROBAMATE |

COX competitive inhibitors of cyclooxygenase; NSAIDs Nonsteroidal Anti-Inflammatory Drugs

**Supplemental Table 4: Contraindicated medications in kidney failure**

| **Characteristic** | **All dialysis participants**  **N=2,248** | **HD**  **N=1,781** | **PD**  **N=467** | **p-value** |
| --- | --- | --- | --- | --- |
| **Medication use based on *drug category*** |  |  |  |  |
| Number of unique drug categories | 0 (0, 0) | 0 (0, 0) | 0 (0, 0) | 0.003 |
| Daily pill burden | 0 (0, 0) | 0 (0, 0) | 0 (0, 0) | 0.003 |
|  |  |  |  |  |
| **Use of contraindicated medications in kidney failure by drug category^a^** |  |  |  |  |
| NSAIDs (other than aspirin) | 106 (4.7) | 94 (5.3) | 12 (2.6) | 0.01 |
| Antiinfectives | 83 (3.7) | 70 (3.9) | 13 (2.8) | 0.24 |
| Psychotherapeutic agents | 32 (1.4) | 27 (1.5) | 5 (1.1) | 0.66 |
| Hypoglycemics | 29 (1.3) | 26 (1.4) | 3 (0.6) | 0.25 |
| Anticoagulants | 13 (0.6) | 11 (0.6) | 2 (0.4) | >0.99 |
| Bisphosphonates | 12 (0.5) | 12 (0.7) | 0 (0) | 0.14 |
| Other | 8 (0.4) | 6 (0.3) | 2 (0.4) | 0.67 |
| Antihyperlipidemics | 4 (0.2) | 4 (0.2) | 0 (0) | 0.59 |
| Anticonvulsants | 1 (0.04) | 1 (0.06) | 0 (0) | >0.99 |
| Antineoplastic | 1 (0.04) | 1 (0.06) | 0 (0) | >0.99 |
| AntiParkinsons | 0 (0) | - | - | - |
| Dementia | 0 (0) | - | - | - |

N (%) or median (IQR).

Data are based on the 120 day period prior to March 31, 2019.

HD hemodialysis; IQR interquartile range; NSAIDs nonsteroidal anti-inflammatory drugs; PD peritoneal dialysis

**^a^** List of specific drugs included for each drug category shown in Supplemental Table 2

**Supplemental Table 5: Potentially inappropriate medication use**

| **Characteristic** | **Dialysis participants aged ≥65y**  **N=1,063** | **HD**  **N=866** | **PD**  **N=197** | **p-value^a^** | **Controls aged ≥65y**  **N=10,599** |
| --- | --- | --- | --- | --- | --- |
| **Use of PIM by drug category^b^** |  |  |  |  |  |
| PPIs | 584 (54.9) | 486 (56.1) | 98 (49.8) | 0.11 | 3,178 (30.0) |
| NSAIDs | 356 (33.5) | 303 (35.0) | 53 (26.9) | 0.03 | 2,088 (19.7) |
| Insulin | 348 (32.7) | 288 (33.3) | 60 (30.5) | 0.45 | 582 (5.5) |
| Antihistamines (1^st^ generation) | 105 (9.9) | 83 (9.6) | 22 (11.2) | 0.50 | 307 (2.9) |
| Benzodiazepines | 78 (7.3) | 67 (7.7) | 11 (5.6) | 0.30 | 675 (6.4) |
| Calcium Channel Blockers | 55 (5.2) | 43 (5.0) | 12 (6.1) | 0.52 | 321 (3.0) |
| Antidepressants | 45 (4.2) | 36 (4.2) | 9 (4.6) | 0.80 | 409 (3.9) |
| Antipsychotics (1^st^ and 2^nd^ generation) | 34 (3.2) | 31 (3.6) | 3 (1.5) | 0.18 | 347 (3.3) |
| Peripheral Alpha-1 Blockers | 34 (3.2) | 33 (3.8) | 1 (0.5) | 0.01 | 86 (0.8) |
| Central Alpha Blockers | 28 (2.6) | 23 (2.7) | 5 (2.5) | >0.99 | 34 (0.3) |
| Antiarrhythmics | 27 (2.5) | 25 (2.9) | 2 (1.0) | 0.21 | 149 (1.4) |
| Bowel Prokinetics | 26 (2.5) | 25 (2.9) | 1 (0.5) | 0.07 | 82 (0.8) |
| Hormones | 21 (2.0) | 13 (1.5) | 8 (4.1) | 0.02 | 586 (5.5) |
| Skeletal | 11 (1.0) | 7 (0.8) | 4 (2.0) | 0.13 | 184 (1.7) |
| Antispasmodics | 6 (0.6) | 6 (0.7) | 0 (0) | 0.60 | 66 (0.6) |
| Non-Benzodiazepine | 6 (0.6) | 6 (0.7) | 0 (0) | 0.60 | 48 (0.5) |
| Androgens | 4 (0.4) | 2 (0.2) | 2 (1.0) | 0.16 | 97 (0.9) |
| Laxatives | 3 (0.3) | 3 (0.3) | 0 (0) | >0.99 | 18 (0.2) |
| Antiinfectives | 1 (0.1) | 1 (0.1) | 0 (0) | >0.99 | 180 (1.7) |
| Antithrombotics | 1 (0.1) | 1 (0.1) | 0 (0) | >0.99 | 17 (0.2) |
| AntiParkinsons | 0 (0) | - | - | - | 6 (0.1) |
| Barbiturates | 0 (0) | - | - | - | 10 (0.1) |
| Opioids | 0 (0) | - | - | - | 5 (0.1) |
| Sulfonylureas | 0 (0) | - | - | - | 39 (0.4) |
| Sympatholytics | 0 (0) | - | - | - | 0 (0) |
| Tranquilizers | 0 (0) | - | - | - | 0 (0) |
|  |  |  |  |  |  |

N (%) or median (IQR).

Data are based on the 120 day period on or prior to March 31, 2019.

HD hemodialysis; IQR interquartile range; NSAIDs nonsteroidal anti-inflammatory drugs; PD peritoneal dialysis; PIM potentially inappropriate medications; PPI proton pump inhibitor; SD standard deviation

^a^ Difference between HD and PD

**^b^** List of specific drugs included for each drug category shown in Supplemental Table 3
